# Effect of Air Oxidation on Texture, Surface Properties and Dye Adsorption of Wood-Derived Porous Carbon Materials

**DOI:** 10.3390/ma12101675

**Published:** 2019-05-23

**Authors:** Suhong Ren, Liping Deng, Bo Zhang, Yafang Lei, Haiqing Ren, Jianxiong Lv, Rongjun Zhao, Xiufang Chen

**Affiliations:** 1 Research Institute of Wood Industry, Chinese Academy of Forestry, Hunan Collaborative Innovation Center for Effective Utilizing of Wood & Bamboo Resources, Beijing 100091, China; rensuh@sina.com (S.R.); rosedlp@sina.com (L.D.); renhq@caf.ac.cn (H.R.); jianxiong@caf.ac.cn (J.L.); 2 Key Laboratory of Bio-based Materials, Qingdao Institute of Bioenergy and Bioprocess Technology, Chinese Academy of Sciences, Qingdao 266101, China; boews@sina.com; 3 Academy of Forestry, Northwest A & F University, Yangling 712100, China; leiyafang@sina.com

**Keywords:** cork, porous structure, air activation, carbon materials, dye adsorbent

## Abstract

Hierarchical porous carbon materials made from cork were fabricated using a facile and green method combined with air activation, without any templates and chemical agents. The influence of air activation on the texture and other surface characteristics of the carbon materials were evaluated by various characterization techniques. Results indicate that air oxidation can effectively improve the surface area and the hierarchical porous structure of carbon materials, as well as increase the number of oxygen-containing functional groups on the carbon surface. The specific surface area and the pore volume of the carbon material activated by air at 450 °C (C800-M450) can reach 580 m^2^/g and 0.379 cm^3^/g, respectively. These values are considerably higher than those for the non-activated material (C800, 376 m^2^/g, 0.201 cm^3^/g). The contents of the functional groups (C–O, C=O and O–H) increased with rising activation temperature. After air activation, the adsorption capacity of the carbon materials for methylene blue (MB) and methyl orange (MO) was increased from 7.7 and 6.4 mg/g for C800 to 312.5 and 97.1 mg/g for C800-M450, respectively. The excellent dye removal of the materials suggests that the porous carbon obtained from biomass can be potentially used for wastewater treatment.

## 1. Introduction

Fast economic and industrial growth has led to the discharge of large volumes of dye wastewater from the rubber, leather, paper, printing and pharmaceutical sectors, among others. This release has intensified to levels hazardous to the environment and human safety. Dye wastewater treatment is necessary for human health and sustainable development. Methods of dye wastewater treatment mainly include adsorption, electrochemical oxidation, addition of chemical thinners, degradation of pollutants via degradation, separation of solid particles via surface filtration and biological techniques [1,2,3,4,5,6,7,8,9,10]. Among these approaches, adsorption is considered a practical method for removing harmful dyes from wastewater owing to its superior performance, cost-efficiency and lowest possible impact on the environment. Because of their large surface area and rich pore structure, as well as surface chemical properties, porous carbon materials have been extensively applied as adsorbent materials to remove dyes in water. Various biomass materials, including pine nut shell, celtuce leaves, coconut shell, lignin, rice husks, cellulose, bamboo, tannin and grape stalk, have thus far been used to fabricate porous carbon materials [11,12,13,14,15,16,17,18,19], exhibiting significant potential as adsorbent materials for removing dye molecules from wastewater.

Recent studies have successfully synthesized a macroporous carbon by using yeast cells [20] and porous carbon fiber monolith by using filamentous fungi [21]. However, the preparation of porous carbon materials via “environmentally friendly” reagents or a completely green process remains a considerable challenge [22,23]. Porous carbon materials are typically produced using hard/soft templates or by chemical activation with massive chemical reagents, requiring a tedious and multiple-step process. This hard-template approach requires a lengthy period to completion and is cost-inefficient and inapplicable for massive production. The soft-template approach relates with the cooperative arrangement of structure-directing agents, which can form liquid crystal phases from appropriate organic carbon precursors but the agents generally entail high costs. For chemical activation, the raw materials or pre-carbonized carbon materials are treated with a large quantity of activating agents such as H_3_PO_4_, NaOH, ZnCl_2_ or KOH. This treatment is followed by high-temperature carbonization to create pore structures and to remove chemical reagents [24,25,26]. The preparation process requires a substantial amount of corrosive chemicals and produces toxic and hazardous wastewater, resulting in various environmental problems.

In addition to textural structure, dye adsorption is influenced by the surface properties of carbon materials, including the contents and varieties of surface functional groups. The regulation of surface functional groups may change the adsorption sites, acidity/basicity and hydrophilicity of the adsorbents, which would adhere organic dyes in wastewater, thus affecting their adsorption performance. The mechanism underlying the adsorption of organic compounds such as methylene blue (MB) primarily occurs by electrostatic interactions, π–π interaction and hydrogen bond [27]. MB is a cationic dye consisting of positively charged nitrogen and sulfur atoms; thus, the negatively charged oxygen-containing functional groups on the surface of the adsorbent can electrostatically interact with the MB molecules. The MB adsorption of carbon materials was affected by both the content of surface functional groups and the average pore diameter of carbon materials. Alatalo et al. [28] suggested that the fructose-based carbon materials with reduced oxygen functionalities can inhibit MB adsorption.

This study mainly aimed to develop a green method for the fabrication of highly efficient biomass-based functional porous carbon. In the present study, raw biomass cork (*Quercus variabilis*) was used as a precursor for the fabrication of functional carbon materials by hydrothermal treatment and post-carbonization. This technique was combined with air activation to create a porous structure and additional oxygen-containing groups. The entire process was facile and completely environment-friendly, requiring no templates or chemicals. The physical and chemical features of the air-activated carbon surface were analyzed by scanning electron microscopy (SEM), transmission electron microscopy (TEM), X-ray diffraction (XRD), N_2_ sorption and X-ray photoelectron spectroscopy (XPS) and other techniques. The multifunctional applications of carbon materials in removing MB and methyl orange (MO) from wastewater were further explored.

## 2. Materials and Methods 

### 2.1. Reagents and Materials

Cork samples collected from Taibai Mountain, Shaanxi Province were dried at room temperature and then ground in a high-speed rotary cutting mill. MB and MO were purchased from Coolaber Science & Technology Co. (Beijing, China). 

### 2.2. Preparation of Functional Porous Carbon Materials

Hierarchically porous carbon was synthesized in three steps: hydrothermal treatment, post-carbonization and air oxidation. First, natural wood samples were hydrothermally treated in stainless steel reactors at 180 °C for 5 h, obtaining hydrothermal carbons (HTCs). These HTCs were then carbonized under the following conditions: atmosphere, N_2_; temperature, 800 °C; N_2_ flow, 100 mL/min; duration: 1 h. This process produced the carbon material C800, which was further activated in static air at 350 °C, 400 °C and 450 °C for 1 h; C800-M350, C800-M400 and C800-M450, respectively, were ultimately obtained.

### 2.3. Characterization

The morphological properties of carbon samples were determined by SEM (S-4800, Hitachi, Tokyo, Japan) and TEM (TF20, Jeol 2100F, Tokyo, Japan). Fourier transform infrared (FTIR) was conducted using a spectrometer (Nicolet FTIR 6700, Thermo Fisher Scientific, Waltham, MA, USA) over a wavenumber in the 400–4000 cm^−1^ range. XRD patterns of carbon samples were generated on a X-ray diffraction diffractometer (D8 Advance, Bruker, Billerica, MA, USA) with CuKa radiation (λ = 1.5147 Å). Data on N_2_ adsorption/desorption were determined using a static volumetric sorption analyzer (ASAP 2020, Micromeritics, Norcross, GA, USA). The surface areas were calculated using the Brunauer-Emmet-Teller (BET) approach. By employing the t-plot technique, the microporous volume of a sample was measured. The pore size distributions were ascertained with non-local density functional theory (DFT). The XPS data were determined on a X-ray photoelectron instrument (ESCALAB 250Xi, Thermo Scientific, Loughborough, UK) with a monochromatized Al Kα line excitation source. Binding energy calibration was performed in accordance with the C1 peak at 284.6 eV. The dye concentration during adsorption tests was measured using a spectrophotometer (UV-2501PC, Shimadzu, Milton Keynes, UK).

### 2.4. Adsorption Testing

The adsorption capacity of the carbon samples was evaluated using MB and MO. Adsorption isotherms were used to determine the adsorption capacity of the samples. About 10 mg the as-prepared wood-derived porous carbon samples were added to 10 mL of the dye solution with different initial concentrations and the suspension was stirred to reach equilibrium for 12 h. For kinetic studies, 10 mg C800-M400 and C800-M450 were used to treat 50 mL of 50 mg/L MB or MO solutions. Owing to slow adsorption, the solution concentrations of C800 and C800-M350 were lower than 10 ppm. For the four carbon samples, 2.0 mL mixtures were collected and then filtered for UV-vis testing for 1–30 min.

## 3. Results and Discussion 

### 3.1. Fabrication of the Functional Carbons

**Figure 1:**
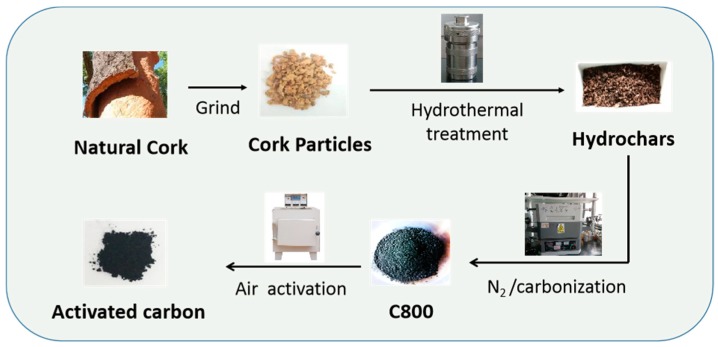
Schematic illustration for preparing functional carbon materials.

A facile and green hydrothermal-carbonization method combined with air activation was used to fabricate functional porous oxygen-rich carbon materials with natural cork as raw material without using any chemical reagents or templates. The corks were first hydrothermally treated with water, which was used as a solvent, to produce hydrochars. Subsequently, post-carbonization of conducted at high temperatures under N_2_ atmosphere to improve the degree of graphitization and the porous structure (Figure 1). The obtained wood-derived carbon material consisted of 89.8 wt% carbon and 6.1 wt% oxygen, as determined by elemental analysis. To generate increased porosity and increase the oxygen-containing functional groups on the carbon materials, the as-prepared carbon was further thermally oxidized in static air at temperature ranging from 350 °C to 450 °C. During air activation, the relatively volatile carbons were partly decomposed and gaseous species such as CO, CO_2_ and hydrocarbons were formed in the presence of oxygen, which were used as an inflating medium to create pores by the carbon reacting with O_2_. Moreover, many pores also can be formed by the removal of carbons. Consequently, functional oxygen-containing species were simultaneously formed using chemical-free thermal oxidation. The results of elemental analysis confirmed that the activated carbons showed increased oxygen content and the functional oxygen groups increased with increasing activation temperature. When C800 (6.1 wt%) was activated at 350 °C, 400 °C and 450 °C, the oxygen content in the functional carbons was increased to 13.8 wt%, 22.4 wt% and 37.3 wt%, respectively.

### 3.2. Surface Morphology of The Functional Carbons

**Figure 2:**
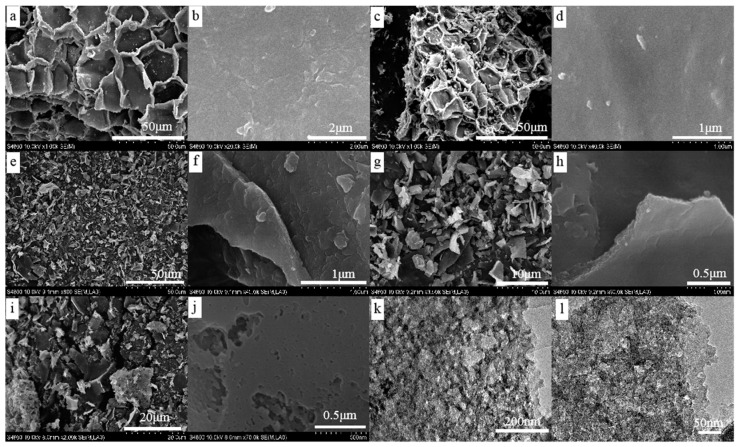
Scanning electron microscope (SEM) images of wood-derived carbon before and after air oxidation (**a**,**b**) raw materials, (**c**,**d**) C800, (**e**,**f**) C800-M350, (**g**,**h**) C800-M400, (**i**,**j**) C800-M450 and transmission electron microscope (TEM) images of C800-M450 (**k**,**l**).

The microstructure of wood-derived porous carbons before and after air oxidation was studied by SEM and TEM (Figure 2). In Figure 2a,b, the raw cork shows a honeycomb-like structure and has obvious folds. After carbonization at 800 °C, the structure was retained and the parts comprising the structure were tightly bound to each other, appearing smooth (Figure 2c,d). After air activation at 350 °C–450 °C, the structural units were separated to form flakes (Figure 2e), which could be ascribed to the degradation of the lignin "“glue” between the cells. The carbon samples exhibited a lamellar structure after air oxidation (Figure 2e–j). The surfaces of the C800-350 and C800-400 samples were relatively smooth and no apparent pore structure was observed at a resolution of 0.5–1 μm (Figure 2f,h). When the oxidation temperature rose to 450 °C, the surface of the carbon material significantly changed and a large number of pores were observed. Figure 2j shows that the C800-M450 sample shows a rough surface with cracks and voids. TEM results (Figure 2k,l) show that C800-M450 displays hierarchical micro/mesoporous structure with a three-dimensionally (3D) interconnected framework. These pores could contribute to the dye adsorption capacity of the carbon samples.

### 3.3. X-ray Diffraction of the Functional Carbons

**Figure 3:**
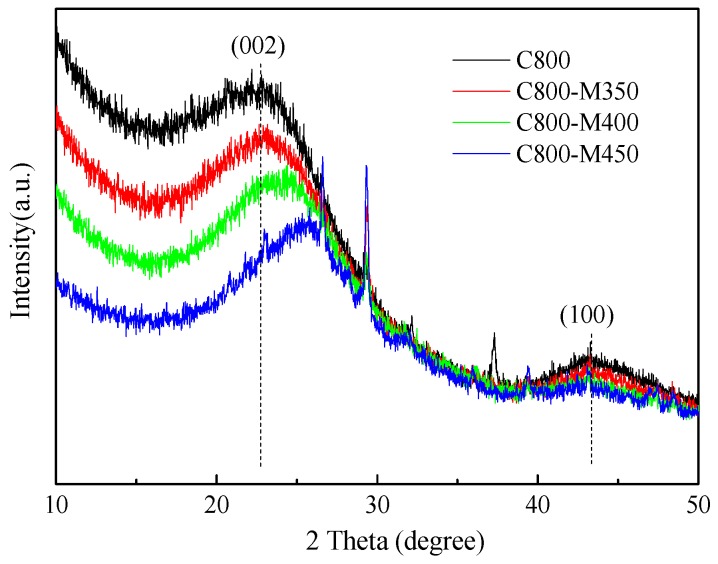
X-ray diffraction (XRD) patterns of wood-derived porous carbons materials.

Figure 3 presents the XRD patterns of wood-derived porous carbons before and after air oxidation. All samples showed a typical broad peak at ~24° and a weak peak at ~43°, corresponding to the (002) and (100) planes of graphitic carbon, respectively [29]. The broad and weak peaks of all samples reflected the low degree of graphitization of carbon samples. Compared with that of the C800 sample, the (002) peak gradually shifted to a higher angle with rising oxidation temperature. This movement suggested the reduction in the d-space of the (002) crystal plane in the graphitic structure after air oxidation. The results implied that the air activation of carbon materials resulted in graphitic structures with improved compactness.

### 3.4. Surface Areas and Pore Size Distribution of the Functional Carbons

**Figure 4:**
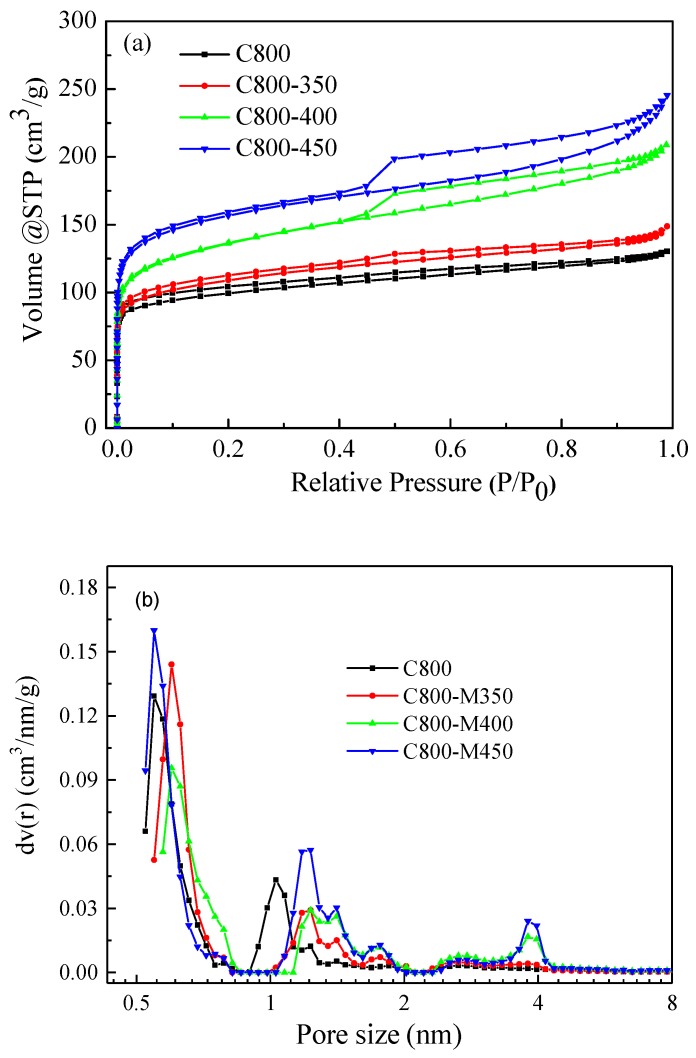
N_2_ adsorption-desorption isotherms (**a**) and density functional theory (DFT) pore size distributions (**b**) of the prepared carbon materials.

Pore texture is one of the significant factors affecting adsorption. The pores of the carbon samples were structurally analyzed using nitrogen adsorption/desorption isotherms. In Figure 4a, all samples show an apparent N_2_ uptake at a low P/P_0_ (<0.1), indicating the presence of a large number of micropores in the porous carbon materials. After air activation, the carbon materials exhibited a markedly higher N_2_ gas adsorption capacity than that of C800 and the gas adsorption capacity gradually increased with rising activation temperature. After air activation, the isotherms of carbon materials were type-IV with an H1 hysteresis loop, suggesting the formation of a mesoporous structure during air activation [30,31]. Nitrogen gas adsorption, to a certain extent, was also observed at a relatively high pressure (>0.8), reflecting the presence of macropores and a large external surface area. C800 had a type I isotherm with no hysteresis loop, indicating that C800 had a predominantly microporous structure. DFT pore size distribution curves confirmed that the pore size distributions of carbon materials after air activation were dominated by micropores (Figure 4b). The carbon materials after air activation had micropores with the pore size in the 0.5–0.8 nm and 1–2 nm ranges; in addition, some mesopores with a radius ranging from 3.2 nm to 4.3 nm, which were generally larger than C800, were also observed. Moreover, the pore size ranging from 1 nm to 2 nm was increased as the air activation temperatures significantly rose. 

The BET surface area, total volume and mesopore volume of the samples—which were measured using N_2_ adsorption–desorption isotherms—are summarized in Table 1. The surface area and pore volume increased with rising activation temperature up to 450 °C, as determined using the BET method. C800-M450 showed the highest BET surface area of 580 m^2^/g and the largest pore volume of 0.379 cm^3^/g. The mesopore volumes also increased sharply with rising activation temperature. The mesopore volume of C800-M450 was almost 3.9 times higher than that of C800, confirming the formation of a mesoporous structure during air activation. It is known that different pore type displays different roles in the dye adsorption performance of carbon materials. A large amount of micropores would offer sufficient space to allow access to the dye molecule transportation. The existence of mesopores would accelerate the kinetic process of dye molecule diffusion. Thus, the improved porous structure might facilitate the penetration and diffusion of dye molecule during adsorption process, probably resulting in stronger adsorption ability to the dye molecules.

### 3.5. FTIR Analysis of the Functional Carbons

**Figure 5:**
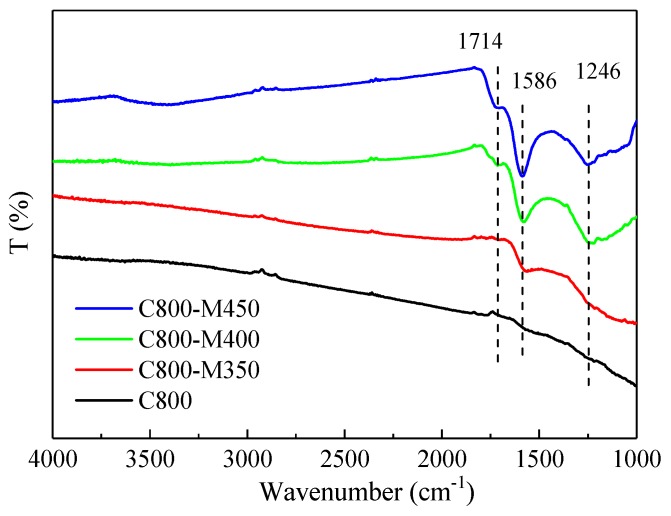
Fourier transform infrared (FTIR) spectra of wood-derived porous carbons materials.

FTIR spectroscopy can present useful data regarding the chemical composition of materials. Figure 5 displays the FTIR spectra of the carbon samples before and after air activation. C800 showed few and weak characteristic bands of oxygen containing groups in the 4000–1000 cm^−1^ region. This finding verified the low content of oxygen-containing groups in C800 (6.1 wt% oxygen content). The carbon samples after air activation showed a broad band near 3400 cm^−1^, which was assigned to the OH stretching vibration of the hydroxyl functional groups (alcohol, phenol or carboxylic acid) [17]. Bands near 1252, 1588 and 1714 cm^−1^ were also observed in the carbon samples after air activation. The first band was attributed to the carboxylate groups (C–O), the middle band was assigned to the aromatic feature (C=O) functionality in the carbon material [32] and the last band was ascribed to C=O for the carbonyl/carboxylic group [28]. Moreover, all bands at 3400, 1252, 1588, 1714 cm^−1^ became stronger when the activation temperature increased, indicating that more oxygen-containing groups were formed during air activation. The elemental analysis and FTIR results were consistent and verified that air activation could effectively increase the surface oxygen-containing functional groups, improving the surface properties of carbon materials. This enhancement would favor for the high adsorption capacity of carbon materials in the dye wastewater.

### 3.6. XPS Analysis of the Functional Carbons

**Figure 6:**
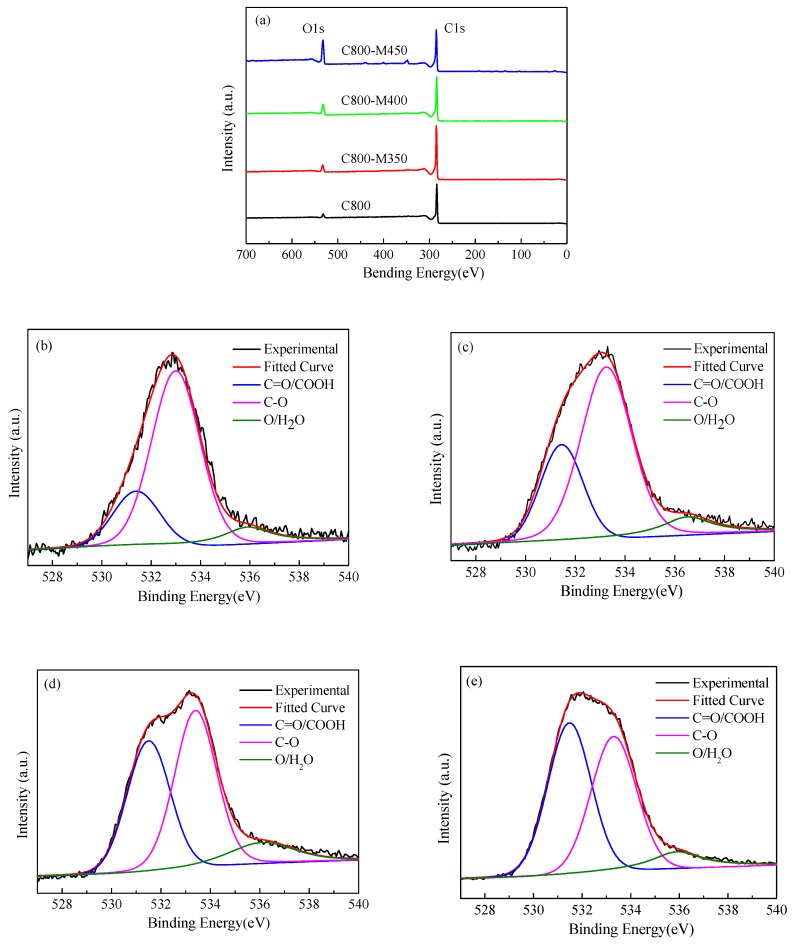
X-ray photoelectron spectroscopy (XPS) spectra of the wood-derived porous carbons materials: (**a**) survey spectra and high-resolution O1s spectra, (**b**) C800, (**c**) C800-M350, (**d**) C800-M400, (**e**) C800-M450.

XPS can be used to analyze the surface chemical characteristics of porous carbon materials. XPS survey spectra of the carbon samples are presented in Figure 6a. The carbon samples before and after activation mainly contained the elements C and O. The C and O contents that were calculated based on XPS data are listed in Table 2. With an increase in activation temperature, the O content increased markedly, whereas the C content decreased. The increase in O content was 5 times from 4.4% (O content in C800) to 21.4% (O content in C800-M450). The C content decreased from about 94.8% (C content in C800) to 77.2% (C content in C800-M450). The results were consistent with those of elemental analysis.

Figure 6b–e present the high-resolution XPS spectra of O 1s of the carbon samples before and after air activation. The O 1s peak could be fitted with the three peaks at 531.4, 533.2 and 535.9 eV, which were attributed to carbonyl (C=O) or ester groups (COOH), singly bonded oxygen (–O–) in the C–O or C–O–R groups and chemisorbed oxygen and water (–O/H_2_O) [33], respectively. Details of the curve-fitting results of the high-resolution XPS spectra for the O 1s region are summarized in Table 2. The contents of the oxygen-containing groups, including C=O/COOH, C–O and –O/H_2_O, increased with rising activation temperature. The C–O group was the predominant group in C800, comprising more than 70% in the O content; by contrast, C=O/COOH and –O/H_2_O only constituted 20% and 10% of the O content, respectively. With an increase in activation temperature, the C=O/COOH content increased the fastest, whereas the –O/H_2_O content increased the slowest. The proportions of C=O/ COOH, C–O and –O/H_2_O in C800-M450 were 47%, 44% and 9%, respectively. The results indicated that air activation preferred the formation of C=O/COOH groups on the surface of the carbon materials. A higher content of C=O/COOH groups suggested the presence of numerous acid functional sites in the carbon materials. Moreover, the functionality can potentially enhance dispensability of carbon materials in water, thereby improving the hydrophilicity of carbon materials and facilitating dye adsorption in wastewater.

### 3.7. Adsorbent Analysis of the Functional Carbons for Methylene Blue/Methylene Orange

**Figure 7:**
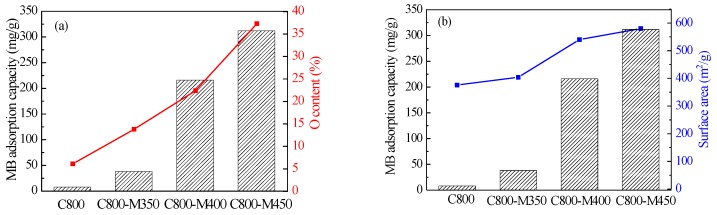
The comparison of MB adsorption capacity, O content (**a**) and surface area (**b**) in the carbon materials before and after air activation.

The adsorption isotherms of MB and MO at room temperature were used to evaluate the adsorption performance of functional carbon materials with dye concentrations varying from 5 mg/L to 300 mg/L. The equilibrium adsorption data were fitted to the Langmuir isotherm model in Equation (1) [34]:

(1) $$\frac{C_{e}}{q_{e}}=\frac{1}{K_{L}q_{0}}+\frac{C_{e}}{q_{0}}$$

where $q_{e}$ is the amount of adsorbate adsorbed per unit mass of the adsorbent (mg/g), Ce is the equilibrium concentration of the solution (mg/L) and $q_{0}$ and K_L_ are the Langmuir constants related to adsorption capacity and rate of adsorption, respectively. The results of the correlation coefficient (R^2^ > 0.98) test. Figures S1 and S2 showed that MB adsorption onto the wood-derived porous carbon is in accordance with the Langmuir isotherm. The adsorption data were also fitted to the Freundlich and Dubinine-Radushkevich models (Table S1) but R^2^ was not good when the experimental data were fitted to these models (Table S2). Thus, Langmuir isotherm model was chosen to analyze the adsorption capacity in this work. The results (Table 3) showed that C800 exhibited poor adsorption of MB (7.7 mg/g) and MO (6.4 mg/g). After air activation, dye adsorption could significantly improve, increasing rapidly with rising activation temperature up to 450 °C. C800-M450 exhibited the highest MB adsorption capacity of 312.5 mg/g (Table 3), which was about 39 times higher than that of C800. The surface area, pore structure and surface property are important factors influencing the dye adsorption capacity of carbon materials. As mentioned previously, air activation of carbon material generated more hierarchical pore structures with a higher surface area and richer surface functional groups. A greater number of pore nanostructures can provide a large number of adsorption sites for dye molecules and improve its adsorption capacity. The rich surface functional groups can facilitate the dispersion of carbon materials in water, bringing the carbon materials in full contact with the dye during adsorption. These properties imparted superior MB adsorption to the functional carbon materials after air activation. Notably, C800 achieved a large surface area with a microporous structure (376 m^2^/g) but exhibited poor adsorption of MB (7.7 mg/g). After air activation at temperatures ranging from 300 °C to 450 °C, the sample exhibited an adsorption capacity 4.8 times that of C800, despite the slight increase in surface area to 404 m^2^/g for C800-M350 as a result of activation. This occurrence suggested that the surface area plays a minor role in improving the adsorption capacity of carbon materials after air activation. The changes in surface properties might primarily influence the adsorption performance of carbon materials after air activation. The results were supported by the correlation of MB adsorption capacity with surface area and oxygen content in the carbon materials affected by air activation temperature (Figure 7). The improved adsorption performance of carbon materials corresponds well with the change trend of oxygen content related to the activation temperature (Figure 7a). Whereas the growth rate of adsorption capacity with rising activation temperature is much higher than that of surface area (Figure 7b).

Moreover, the adsorption performance of C800-M450 toward MO was 97.1 mg/g, about 15.2 times higher than that of C800 (6.4 mg/g). Comparison of the adsorption data reveals that C800-M450 exhibits an adsorption capacity of MB 3 times higher than that of MO, which could have resulted from the large number of oxygen-containing groups of the adsorbent surface [35,36], large surface area and strong hydrogen bonding and electronic attraction, such as π–π stacking interactions between aromatic rings of MB and aromatic skeletons on the surface [37]. Compared to those of most other biomass-derived carbon materials (Table S3), C800-M450 had a relative high adsorption performance. It was worthy to note that most crude biomass-derived carbons reported previously were fabricated by chemical activation with a large amount of chemical reagents to obtain porous carbon with a large surface area. In this work, the functional carbons were fabricated using a facile and green method, combined with air activation, without any templates and chemical agents. The synthetic method has the advantages of simple, sustainable and easy large-scale production.

The influence of pH of MB solution on the adsorption capacity by C800-M450 sample was studied at 298 K in the range from 4 to 10. The pH of MB solution was adjusted by using 0.1 mol/L NaOH solution or 0.1 mol/L HCl solution. The MB adsorption capacity of C800-M450 from the aqueous solution at pH = 4, 7, 10 was 296 mg/g, 312 mg/g and 298 mg/g, respectively. The MB adsorption capacity slightly increased with increasing pH from 4 to 7, while the adsorption capacity slightly decreased with increasing pH from 7 to 10. The slight decrease in the adsorption capacity at pH = 4–10 also suggested the high level of pH tolerance of the obtained functional carbons for MB adsorption, which would be extremely favorable for practical applications. The effect of temperature of MB solution on the adsorption capacity by C800-M450 sample was also investigated at 298–423 K. The adsorption capacity increased with increasing the adsorption temperature. The MB adsorption capacity of C800-M450 from the aqueous solution at 298 K, 413 K, 423 K was 312 mg/g, 348 mg/g and 382 mg/g, respectively. The result indicated that a higher temperature would facilitate the high adsorption performance.

The kinetics of adsorption describes the rate of adsorption at the solid–liquid interface. For the analysis of MB and MO adsorption kinetics, experimental data were modeled using pseudo-first order function (Equation (2)) and pseudo second- order function Equation (3), hence the simulation of the process. Equations (2) and (3) can be expressed as follows:

(2) $$\ln(q_{e}-q_{t})=\ln q_{e}-\frac{k_{1}t}{2.303}$$

(3) $$\frac{t}{q_{t}}=\frac{1}{k_{\mathrm{ad}}{\;q}_{e}^{2}}+\frac{t}{q_{e}}$$

where $q_{e}$ and q_t_ denote the adsorption capacity (mg/g) at equilibrium and t time, respectively; k_1_ represents the pseudo-first order rate constant (min^−1^); $k_{\mathrm{ad}}$ is the pseudo-second-order rate constant (g/mg/min). The results of the regression coefficient (R^2^) test were shown in Table 4 and Table 5 and Figures S3 and S4. The R^2^ values derived from the first-order kinetic model were 0.8467–0.9515, while the R^2^ values derived from the second-order kinetic model were greater than 0.99. A higher fit between the experimental data and the pseudo-second order model was obtained. Thus, the pseudo-second order model was more suitable to describe the adsorption kinetics of MB and MO on the as-prepared carbons. The result indicated that the adsorption mechanism depends on the concentration of adsorbate and adsorbent. The experimental values of $q_{e}$ agreed with the $q_{e}$ values calculated using the pseudo-second order model for C800-M450 and C800-M400. These results demonstrated that MB and MO adsorption was controlled by chemisorption [3]. The q_e_ values for C800-M450 and C800-M400 are considerably larger than the values for C800, confirming that dye adsorption is influenced by surface properties, including surface area and surface functional groups.

Reusability of carbon material is important for effective practical application in the wastewater treatment. The cyclic adsorption behavior of C800-M450 was also investigated. 30 mg of C800-M450 sample was added into 30 mL of MB solution (500 mg/L) in a batch container. After completion of the adsorption reaction, the carbon sample was removed from the mixture by centrifugation, washed with water and ethanol for several times to remove the adsorbed MB in the carbon sample thoroughly, dried at 50 °C for 12 h and then reused directly without other treatment. The results showed that the C800-M450 could be reused four times with high adsorption capacity retention of 85% after four cycles.

## 4. Conclusions

In summary, we propose a template-free method for the fabrication of hierarchical porous carbon with a large surface area and numerous oxygen-containing groups by using natural wood as a carbon precursor without any templates or chemical activation. Air oxidation at 450 °C can result in a large surface area of 580 m^2^/g. Air activation can effectively improve the surface properties of carbon materials and functional oxygen-containing groups increased in content with rising activation temperature. After air activation, the adsorption capacity of carbon materials for MB and MO increased from 7.7 mg/g to 312.5 mg/g for C800 and from 6.4 mg/g to 97.1 mg/g for C800-M450. The superior adsorption could be attributed to the enhanced pore structure and surface properties (e.g., surface functional oxygen-containing groups). The surface area plays a minor role in improving the adsorption capacity of carbon materials after air activation, whereas the changes in surface properties primarily influence the adsorption performance of carbon materials after air activation. This modest technique exhibits potential for wide application in the production of porous functional carbon materials with a large surface area by using other types of raw biomass, such as bamboo and hard wood. With hierarchical pores and superior surface property, these materials can be suitable for adsorption, energy storage and conversion, as well as catalysis.

## Figures and Tables

**Table 1:** Pore structure parameter of carbon materials.

Sample	S_BET_ (m^2^/g)	V_total_ (cm^3^/g)	V_meso_ (cm^3^/g)	Pore Size (nm)
C800	376	0.201	0.043	0.55/1.03
C800-M350	404	0.232	0.059	0.60/1.22/3.80
C800-M400	540	0.325	0.136	0.60/1.23/3.84
C800-M450	580	0.379	0.167	0.55/1.23/3.86

S_BET_: BET surface area, V_total_: total pore volume, V_meso_: mesopore volume.

**Table 2:** Carbon and oxygen content determined by XPS data and results of the curve-fitting of the high-resolution XPS spectra for the O 1s region.

Sample	C 1s (%)	O 1s (%)	O 1s C=O/COOH (%)	O 1s C–O (%)	O 1s –O/H_2_O (%)
C800	94.8	4.4	0.9	3.1	0.4
C800-M350	92.7	6.6	1.8	4.2	0.6
C800-M400	88.5	10.8	4.3	5.2	1.3
C800-M450	77.2	21.4	10.1	9.3	1.9

**Table 3:** The Langmuir isotherm parameters for the adsorption of organic dyes onto as-prepared carbons.

Sample	Dye	K_L_ (L/mg)	$q_{0}\;(\mathrm{mg}/g)$
C800	MB	0.2089	7.7
C800-M350	MB	0.1139	38.6
C800-M400	MB	1.2105	217.4
C800-M450	MB	0.2353	312.5
C800	MO	1.1422	6.4
C800-M450	MO	0.4813	97.1

**Table 4:** Regression results from pseudo-first-order kinetic model.

Samples	Dye	$q_{e}\;(\mathrm{mg}/g)$	$k_{1}\;(1/\min)$	R^2^
C800	MB	6.9	0.0141	0.9060
C800-M350	MB	14.1	0.1555	0.9157
C800-M400	MB	162.9	0.1426	0.9515
C800-M450	MB	220.2	0.0482	0.9091
C800	MO	5.0	0.1211	0.9449
C800-M450	MO	77.9	0.0943	0.8467

**Table 5:** Regression results from pseudo-second-order kinetic model.

Sample	Dye	$q_{e}\;(\mathrm{mg}/g)$	k_ad_ (g/mg/min)	R^2^
C800	MB	8.1	0.2287	0.9992
C800-M350	MB	39.1	0.0168	0.9949
C800-M400	MB	189.1	0.0010	0.9912
C800-M450	MB	200.0	0.0019	0.9964
C800	MO	6.5	0.0250	0.9995
C800-M450	MO	90.9	0.0010	0.9964

## Supplementary Materials

The following are available online at https://www.mdpi.com/1996-1944/12/10/1675/s1, Figure S1: q_e_ vs C_e_ plots of as-prepared carbons: (a) MB and (b) MO. Figure S2: Isothermal adsorption of (a) C800-M450−MB, (b) C800-M400−MB, (c) C800-M350−MB, (d) C800−MB, (e) C800-M450−MO, (f) C800−MO, fitted with the Langmuir model. Figure S3: Translated kinetic adsorption ln(q_e_-q_t_) vs t plots of (a) C800-M450−MB, (b) C800-M400−MB, (c) C800-M350−MB, (d) C800−MB, (e) C800-M450−MO, (f) C800−MO, fitted with pseudo-first-order model. Figure S4: Translated kinetic adsorption t/q_t_ vs t plots of (a) C800-M450−MB, (b) C800-M400−MB, (c) C800-M350−MB, (d) C800−MB, (e) C800-M450−MO, (f) C800−MO, fitted with pseudo-second-order model. Table S1: Lists of adsorption isotherm models. Table S2: Parameters of the Freundlich and Dubinine-Radushkevich models for adsorption of MB/MO on the carbons. Table S3: Comparison of adsorption performance of various carbon-based adsorbent materials.
