# Supplementary material for: Effect of Air Oxidation on Texture, Surface Properties and Dye Adsorption of Wood-Derived Porous Carbon Materials
Source: Materials (Basel). 2019 May 23;12(10):1675. doi: 10.3390/ma12101675 (PMC6566616; doi:10.3390/ma12101675)
Supplement: Supplementary file 1 [file materials-12-01675-s001.pdf]

materials

Supplementary Materials

Effect of Air Oxidation on Texture, Surface Properties, and Dye Adsorption of Wood-Derived Porous Carbon Materials

Suhong Ren [1], Liping Deng [1], Bo Zhang [2], Yafang Lei [3], Haiqing Ren [1], Jianxiong Lv [1], Rongjun Zhao [1,*] and Xiufang Chen [2,*]

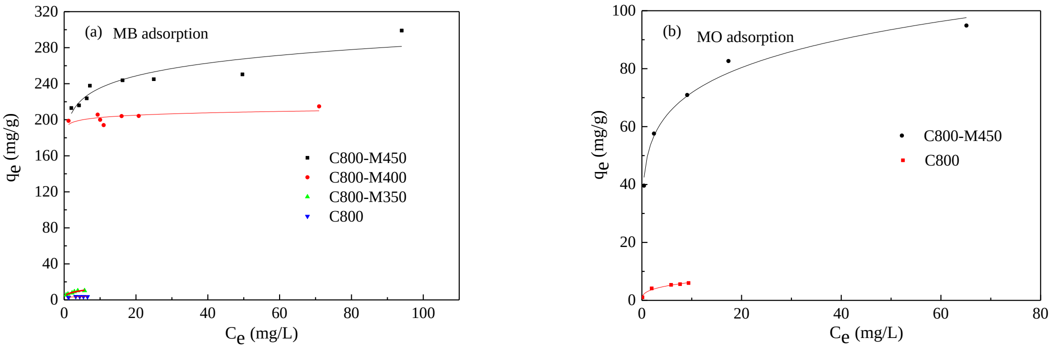

Figure S1. q_e vs C_e plots of as-prepared carbons: (a) MB and (b) MO.

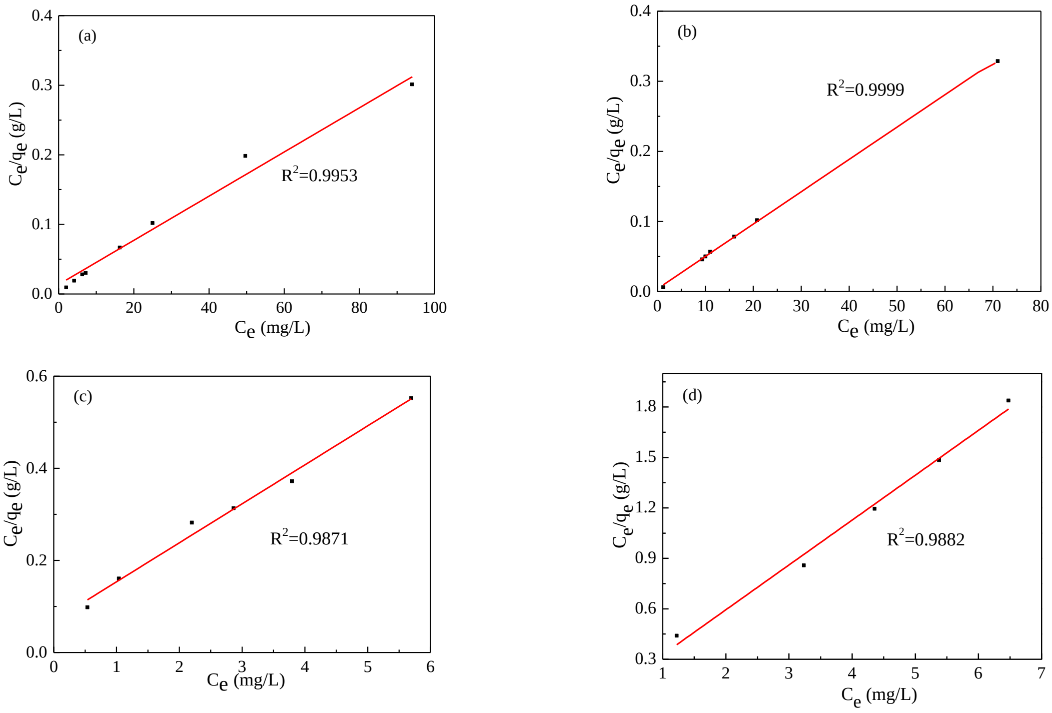

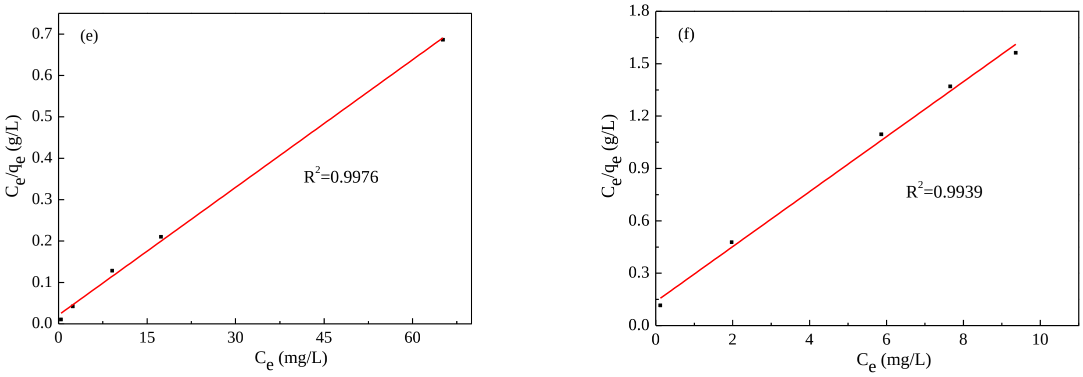

Figure S2. Isothermal adsorption of (a) C800-M450−MB, (b) C800-M400−MB, (c) C800-M350−MB, (d) C800−MB, (e) C800-M450−MO, (f) C800−MO, fitted with the Langmuir model.

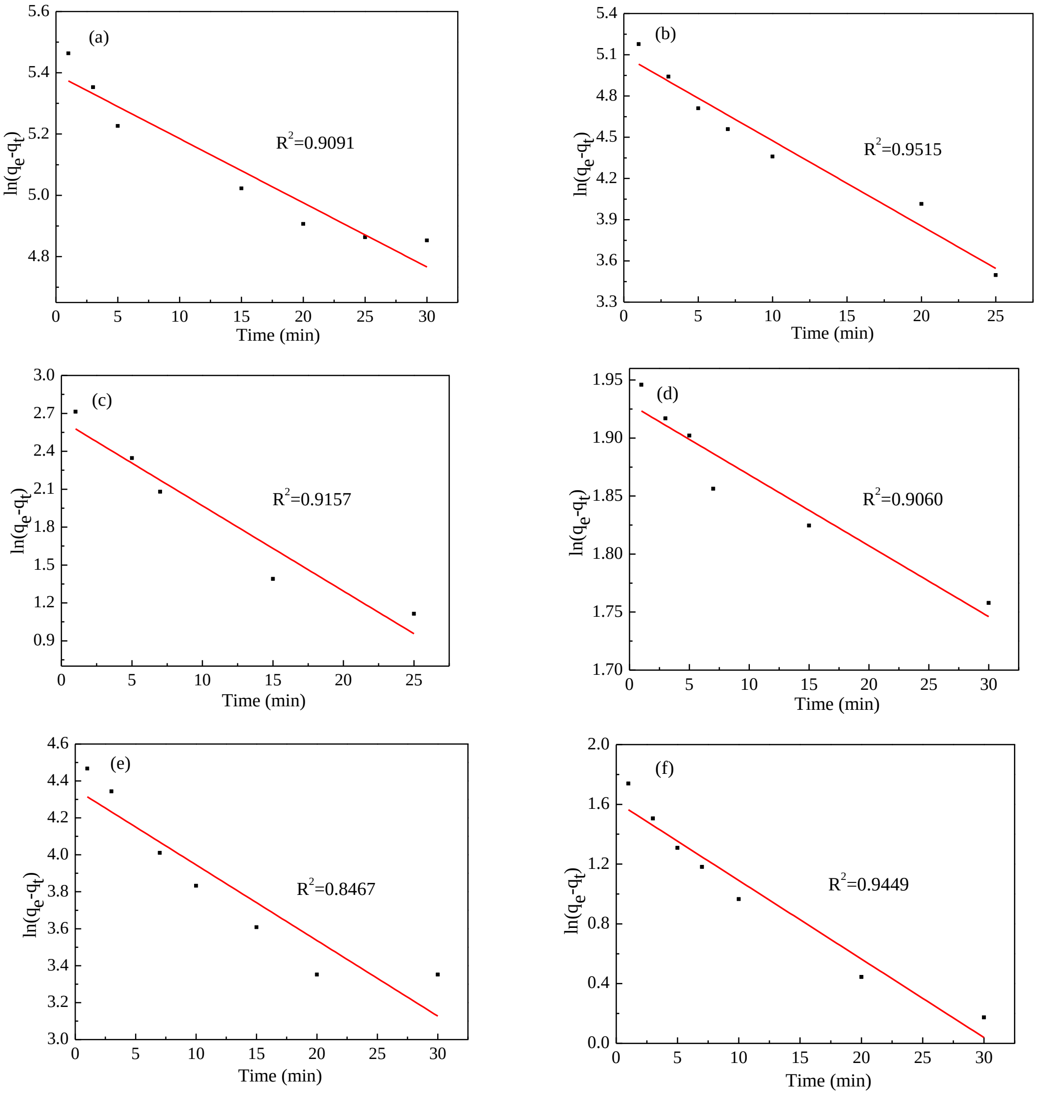

Figure S3. Translated kinetic adsorption ln(q_e-q_t) vs t plots of (a) C800-M450−MB, (b) C800-M400−MB, (c) C800-M350−MB, (d) C800−MB, (e) C800-M450−MO, (f) C800−MO, fitted with pseudo-first-order model.

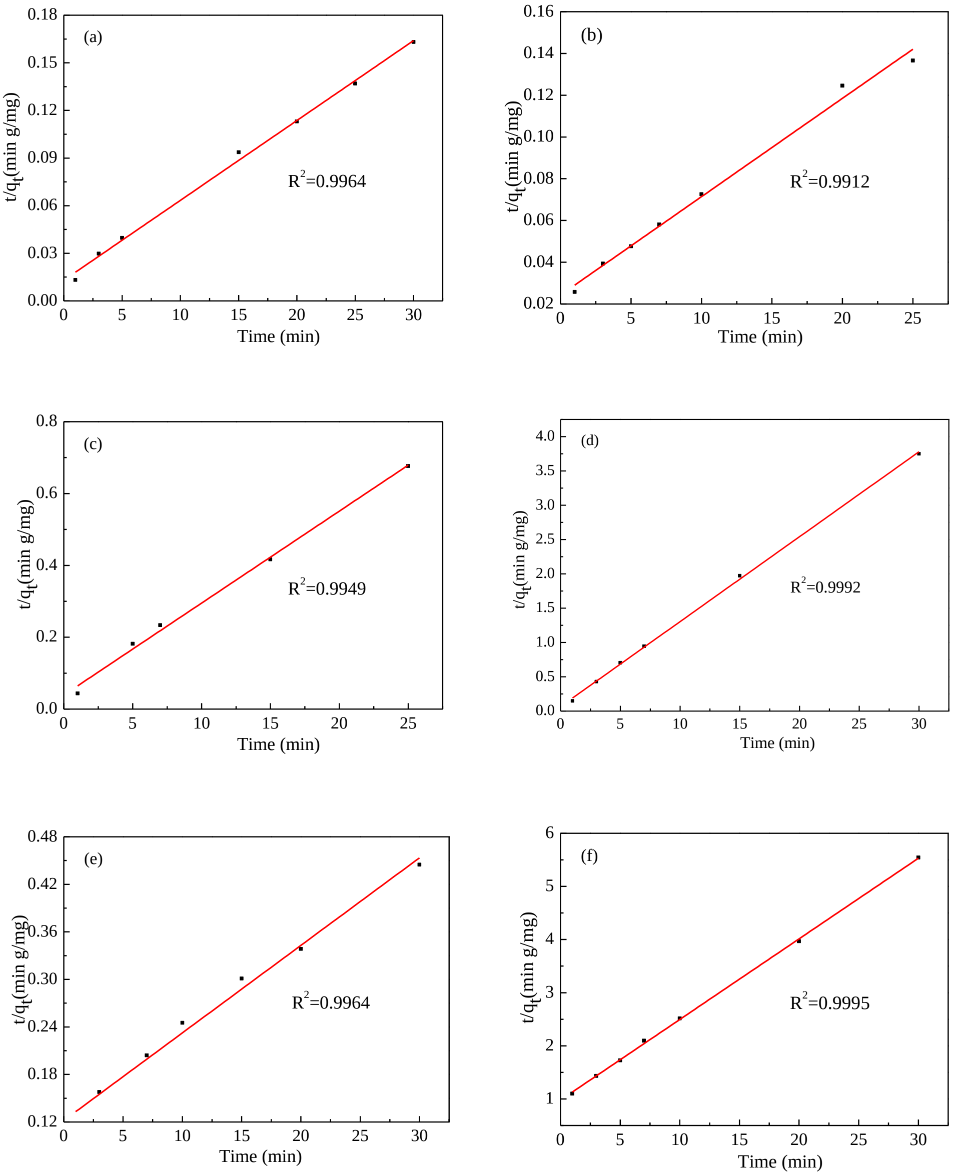

Figure S4. Translated kinetic adsorption t/q_t vs t plots of (a) C800-M450−MB, (b) C800-M400−MB, (c) C800-M350−MB, (d) C800−MB, (e) C800-M450−MO, (f) C800−MO, fitted with pseudo-second-order model.

Table S1. Lists of adsorption isotherm models.

Isotherm	Linear form	Plot
Freundlich	$\ln q_e = \ln K_F + \frac{1}{n} \ln C_e$	$\ln q_e$ vs $\ln C_e$
Dubinin-Radushkevich	$\ln q_e = \ln q_m - k_d \varepsilon^2$	$\ln q_e$ vs ε^2

Table S2. Parameters of the Freundlich and Dubinine-Radushkevich models for adsorption of MB/MO on the carbons.

Samples	Dye	Freundlich			Dubinin-Radushkevich		
		K_F ((mg/g) (mg/L)^n)	1/n	R^2	k_d (mol^2/kJ^2)	q_m (mg/g)	R^2
C800	MB	2.81	0.1603	0.9672	2.00E-07	4.48	0.3477
C800-M350	MB	6.51	0.288	0.9959	1.00E-07	12.89	0.3573
C800-M400	MB	196.37	0.0154	0.8457	8.00E-07	209.94	0.3868
C800-M450	MB	204.94	0.0555	0.8793	1.00E-07	239.87	0.6075
C800	MO	2.61	0.4033	0.9783	5.00E-08	5.32	0.9734
C800-M450	MO	47.80	0.1757	0.9860	7.00E-08	76.72	0.7622

Table S3. Comparison of adsorption performance of various carbon-based adsorbent materials.

Raw materials	Dye	Surface area of carbon (m^2/g)	q_e (mg/g)	reference
Cork	MB<br>MO	580	312<br>96	This work
Camellia oleifera seed shell	MB	1882	493	37
Banana peel	MO	24	21	[1]
Orange peel	MO	24	21	
Poplar catkins	MO	351	154	[2]
Rattan	MB	1135	359	[3]
Cotton stalk	MB	795	194	[4]
Sunflower stalk	MB	1.2	205	[5]
Tomato processing waste	MB	1093	400	25

References

1. Annadurai, G.; Juang, R.S.; Lee, D.J. Use of cellulose-based wastes for adsorption of dyes from aqueous solutions. *J. Hazard. Mater.* **2002**, *92*, 263–274.
2. Liu, X.; Sun, J.; Duan, S.X.; Wang, Y.N.; Hayat, T.; Alsaedi, A.; Wang, C.M.; Li, J.X. A valuable biochar from poplar catkins with high adsorption capacity for both organic pollutants and inorganic heavy metal Ions. *Sci. Rep.* **2017**, *7*, 10033.
3. Islam, M.A.; Ahmed, M.J.; Khanday, W.A.; Asif, M.; Hameed, B.H. Mesoporous activated carbon prepared from NaOH activation of rattan (*Lacosperma secundiflorum*) hydrochar for methylene blue removal. *Ecotox. Environ. Safe.* **2017**, *138*, 279-285.
4. Deng, H.; Yang, L.; Tao, G.H.; Dai, J.L. Preparation and characterization of activated carbon from cotton stalk by microwave assisted chemical activation-Application in methylene blue adsorption from aqueous solution. *J. Hazard. Mater.* **2009**, *166*, 1514-1521.
5. Sun, G.; Xu, X. Sunflower stalk as adsorbents for color removal from textile wastewater. *Ind. Eng. Chem. Res.* **1997**, *36*, 808–812.

© 2019 by the authors. Submitted for possible open access publication under the terms and conditions of the Creative Commons Attribution (CC BY) license (http://creativecommons.org/licenses/by/4.0/).
